# Health economic evaluation of health promotion and disease prevention in Germany: a scoping review

**DOI:** 10.1186/s13561-026-00859-0

**Published:** 2026-09-25

**Authors:** Eva Juliane Beer, Michaela Kirschneck, Michaela Coenen, Rosa Maria Sija Visscher

**Affiliations:** 1 Institute for Medical Information Processing, Biometry, and Epidemiology (IBE), LMU Medizin, LMU Munich, Elisabeth-Winterhalter-Weg 6, Munich, 81377 Germany; 2 Pettenkofer School of Public Health, Munich, Germany; 3 Careum School of Health, Part of the Kalaidos University of Applied Sciences, Gloriastrasse 18a, Zurich, 8006 Switzerland

**Keywords:** Disease prevention, Health promotion, Health-economic evaluation, Cost-effectiveness, Scoping review, Health economics

## Abstract

**Background:**

Health promotion and disease prevention are receiving increasing attention in Germany because of their potential to improve population health and reduce healthcare costs. Health-economic evaluations (HEEs) are essential for informing evidence-based decision-making in this field. However, HEEs of preventive interventions often face methodological challenges. This scoping review examines the current state and characteristics of HEEs conducted in the context of health promotion and disease prevention in Germany.

**Methods:**

A scoping review was conducted to map the landscape of HEE of health promotion and disease prevention interventions in Germany, following a priori–registered protocol. Three bibliographic databases (MEDLINE, EMBASE and Web of Science) were searched from their inception to March 2025. The screening of titles, abstracts, and full texts was performed in a blinded, double-reviewer, and stepwise manner using predefined inclusion criteria: (i) focus on the German population, (ii) application of an HEE as the primary research method, and (iii) evaluation of a health-promoting or disease-preventing intervention. Studies focusing on tertiary prevention were excluded.

**Results:**

Of the 4,632 records identified, 162 eligible original studies met the inclusion criteria. The volume of published HEEs has increased over time. Most studies (53.7%) assessed primary preventive interventions. Cost-effectiveness analysis was the most frequently applied HEE analysis type. Incremental cost-effectiveness ratios (ICERs) are commonly reported as incremental costs per quality-adjusted life year (QALY) gained (42.4% of all ICERs). In 85.2% of the studies, the intervention was judged to be cost-effective. A model-based approach was used in 75.9% of the studies, and 81.3% of these studies relied partly on non-German data sources. More than half of all studies (56.1%) reported limitations due to a lack of German-specific data.

**Conclusions:**

HEEs related to health promotion and disease prevention interventions in Germany exhibit substantial methodological heterogeneity. Frequent reliance on non-German data and the large proportion of interventions deemed cost-effective may reflect contextual data gaps and potential publication bias. These issues complicate the interpretation of economic evidence and pose challenges for decision-makers seeking to prioritize preventive strategies.

**Trial registration:**

https://osf.io/h6749/overview

**Supplementary Information:**

The online version contains supplementary material available at https://doi.org/10.1186/s13561-026-00859-0.

## Background

Health promotion and disease prevention are essential pillars of modern public health systems. Health promotion seeks to enable individuals and communities to increase control over, and to improve, their health [1]. Although achieving health equity is not intrinsic to the definition of health promotion, both health promotion and disease prevention play a crucial role in fostering equitable health outcomes. Moreover, disease prevention seeks to reduce the occurrence, progression or impact of diseases through targeted interventions [2]. In Germany, the importance of health promotion and disease prevention has been increasingly recognized, particularly following the implementation of the Preventive Health Care Act (Präventionsgesetz) in 2015. This legislative framework reinforced the role of statutory health insurance providers in funding and implementing preventive interventions and emphasized the need for intersectoral cooperation to foster health equity and reduce avoidable disease burdens.

However, as health care resources are limited, prioritizing effective and efficient interventions becomes crucial. Health-economic evaluations (HEEs) serve as systematic tools to inform such decisions [3]. They compare the costs and outcomes of alternative strategies [3] and provide evidence on the value for money of interventions, therapies, pharmaceuticals and similar measures [4]. Unlike clinical interventions, preventive strategies often involve long-term time horizons, diffuse benefits, and broader societal impacts, which pose methodological challenges in evaluation [5, 6]. The demographic shift in Germany, characterized by an aging population and low birth rates [7], together with increasing chronic disease prevalence [8] and economic constraints [9] underscore the need for strategic public health planning. Robust health-economic evidence is essential to guide sustainable investment in health promotion and disease prevention. To guide policymakers and stakeholders in making informed decisions, it is crucial to understand the various methodological approaches available for conducting HEEs and their specific applications.

There is a variety of methodological approaches to conduct HEEs. There are Cost-Minimization Analyses (CMAs) which address the question of finding a cost-saving alternative by conducting separate cost analyses of two or more alternative interventions on the condition that the evaluation of the various interventions is conducted within the same context and the outcomes of the intervention are equal [10]. Cost-Consequence Analyses (CCAs) present costs and a range of relevant outcomes in a disaggregated manner, allowing decision‑makers to consider multiple consequences simultaneously without aggregating them into a single summary measure [11]. Although CCAs do not provide a single decision rule, they are frequently applied in public health and prevention contexts where interventions affect multiple outcomes. Cost-Benefit Analyses (CBAs) represent a classical form of economic evaluation in which both the costs and the consequences of alternative interventions are expressed in monetary terms [3]. Given that the clinical outcomes of competing interventions are often not identical [10], Cost-Effectiveness Analyses (CEAs) enable the comparison of effects that are not easily monetized as measures in natural units [5]. A specific form of CEAs are Cost-Utility Analyses (CUAs), which expresses health outcomes in standardized utility units to facilitate comparisons across diverse health care interventions [3]. The most widely used metric is the concept of the quality-adjusted life year (QALY) [10], which integrates the effects of reduced mortality and morbidity while reflecting patient preferences [3, 11].

To assess cost-effectiveness, CEAs and CUAs commonly report an incremental cost-effectiveness ratio (ICER), defined as the additional cost required to gain one additional unit of effect [3]. Cost‑effectiveness can also be assessed by comparing the calculated ICER to a cost‑effectiveness threshold, which may be derived either from willingness‑to‑pay considerations or from opportunity costs in terms of forgone health benefits from alternative uses of resources. In the German context, the opportunity cost perspective is particularly relevant, as healthcare resources are constrained and allocation decisions necessarily involve trade‑offs rather than explicit willingness‑to‑pay threshold. If the ICER is less than this threshold, the intervention is deemed cost-effective [3]. As even the most favorable cost-effectiveness ratio is of limited value without the financial means for implementation, Budget Impact Analyses (BIAs) are necessary to assess the affordability and financial feasibility of interventions deemed cost-effective. BIAs assess the direct financial consequences associated with the reimbursement of health technology within a specific healthcare system [12]. BIAs are not intended to replace CEAs or CUAs but rather to complement them [12].

Evidence-based recommendations and quality criteria for HEEs in the international context are offered by the reporting framework of the Consolidated Health Economic Evaluation Reporting Standards (CHEERS) [13] as well as by the International Society for Pharmacoeconomics and Outcomes Research (ISPOR) Task Force [14] with ongoing Good Practice Reports [15, 16]. The National Institute for Health and Clinical Excellence (NICE) of the UK provides recommendations on QALY-based CUAs [17]. In the German context, methodological recommendations of the Institute for Quality and Efficiency in Health Care (IQWiG), with a focus on IQWiG-own benefit evaluation methods [18], exist. The Standing Committee on Vaccinations (STIKO) offers guidance for modeling approaches to vaccinations [19], whereas the Hanover Consensus provides detailed recommendations on methods for HEEs in Germany [20]. Despite the availability of general methodological or intervention-specific guidance for health economic evaluations, it remains unclear how HEEs of health promotion and disease prevention interventions are currently conducted and reported in practice within the German context. This lack of transparency limits the ability of researchers, policymakers, and guideline developers to understand prevailing methodological approaches, identify common challenges, and assess the decision relevance of the existing evidence base.

To date, no prior attempts have been identified that systematically mapped and categorized HEEs of health-promoting or disease-preventing interventions within the German context. Although one scoping review examined methodological issues across reviews of economic evaluations of disease prevention and health promotion but did not consider German-specific evidence of frameworks [21]. In addition, a book contribution aimed to describe the status quo of HEEs in health promotion and prevention in Germany; however, its analysis was simplified and time-restricted and did not follow a systematic review methodology [6]. As a result, a comprehensive and up-to-date overview of the scope, characteristics, and methodological practices of HEEs informing health promotion and disease prevention in Germany has been lacking. Therefore, the aim of this scoping review was to systematically map and categorize HEEs conducted for health promotion and disease prevention interventions in Germany to support further research in this field.

## Methods

To provide a comprehensive overview of the existing landscape, a scoping review was considered to be the most suitable type of review. The work was conducted in accordance with the methodology of the Joanna Briggs Institute (JBI) for scoping reviews [22], ensuring methodological rigor. The reporting adhered to the PRISMA Extension for Scoping Reviews (PRISMA-ScR) Checklist and Explanation [23] (Additional file 1). Furthermore, the review process followed a preregistered protocol published online in the open science framework [24].

### Search strategy

The development of the search string followed the “PCC” method (population, concept, context) [22], as recommended for scoping reviews since as it supports broad research questions and facilitates the identification, mapping, and categorization of heterogeneous evidence without restricting the review to predefined interventions, comparators, or outcomes. Hence, this review focused on the German population. The concept used was health-economic evaluations, and the context applied was interventions regarding health promotion and disease prevention. The initial string was conducted by the first author and subsequently refined through team discussions with experienced researchers. To validate the search, 10 relevant articles were identified by the first author and used to adjust the query string. The search was restricted to articles published in German or English, and only completed primary studies were considered; study protocols and systematic reviews were excluded. The reference lists of relevant systematic reviews and included studies were additionally screened for other relevant primary studies. No restriction on the year of publication was applied. Three bibliographic databases (Ovid Medline, Ovid Embase and Clarivate Web of Science) were searched from their inception to March 11, 2025. These databases were selected because they provide broad and complementary coverage of biomedical, public health, and health services research and are commonly used in health technology assessment and public health decision‑making contexts. The query string used for Ovid Medline is displayed in Table 1 and was adapted for each database to account for differences in indexing and search functionality (Additional file 2).

**Table 1:** Search string used for Ovid Medline

Aspect	String
Basics	(humans) [MeSH terms] AND
Population	(germany) [MeSH terms] AND
Concept	(economic* OR cost*) [Abstract, Title] adj2 (evaluation OR calculation OR analys* OR comparison OR health care). [Abstract, Title] OR (“health economics” [Multi-purpose] OR “return on investment” [Multi-purpose] OR “incremental cost-effectiveness ratio” [Abstract, Title] OR “ICER” [Abstract, Title] OR “quality-adjusted life years” [Abstract, Title, MeSH terms] OR “QALY” [Abstract, Title] OR “budget-impact analysis” [Abstract, Title] OR “cost-minimization analysis” [Multi-purpose] OR “cost-of-illness analysis” [MeSH terms] OR “cost-utility analysis” [Abstract, Title] OR exp “costs and cost analysis”/ OR “cost-benefit analysis” [MeSH terms] OR “cost-effectiveness analysis” [MeSH terms] AND
Context	(promotion OR program* OR prevent* OR campaign OR education OR precaution OR public OR rehab*) [Abstract, Title] OR (*"health promotion”/ OR *"preventive medicine”/ OR “universal prevention” [Multi-purpose] OR *"primary prevention”/ OR “vaccination” [MeSH terms] OR *"secondary prevention”/ OR “screening” [Abstract, Title] OR *"tertiary prevention” OR *"rehabilitation”/)

*MeSH *Medical Subject Headings

### Eligibility criteria

According to the PCC framework, the inclusion and exclusion criteria shown in Table 2 were applied for the assessment of eligibility. The definition of HEEs was based on the principle that full economic evaluations involve a comparative analysis of alternative interventions that considers both their costs and consequences [3]. CMAs were included only when they explicitly compared costs between alternatives under the stated assumption of equivalent outcomes. In this scoping review, primary prevention was defined as all measures taken before the initial manifestation of a disease to prevent its onset [25]. Secondary preventive measures were defined as interventions aimed at early detection and containment of disease, where the pathological process has already begun [25]. Tertiary prevention applied when a disease or adverse condition has already manifested, with a focus on managing and mitigating its impact [25]. Due to difficulties in the precise delineation of definitions, studies addressing tertiary prevention, rehabilitation and disease management were excluded. During the training and pilot screening phase, substantial overlap between tertiary prevention, early treatment, and disease management approaches was observed, often compounded by limited or unclear reporting of intervention objectives. As a result, a consistent and reproducible classification of such interventions was not feasible within the scope of this review. Additionally, pharmacological interventions were not included, as they represent a special and well-researched field.

**Table 2:** Eligibility criteria

Inclusion criteria	
Population	Fully or partially German
Concept	Full health-economic evaluations
Context	Health promotion, primary and secondary prevention
Exclusion criteria	
Population	Non-German
Concept	Non comparing health-economic evaluations Reported monetary outcomes other than in Euro or Deutsche Mark
Context	Tertiary prevention and rehabilitation Disease management Pharmacological prevention

### Study selection

The search results were exported to EndNote [Version 21; Clarivate; Philadelphia, PA, USA] and uploaded to the web-based application Rayyan [Version 2023; Rayyan Systems Inc.; Cambridge, MA, USA]. Duplicates were removed via the automatic identification function of the Rayyan application and confirmed by hand by one of the reviewers. Two training courses regarding the different types of HEEs were conducted to bring all researchers to the same level of knowledge. In the pilot phase, identical abstracts were screened by all researchers, followed by a stepwise procedure, in which all six authors screened the titles/abstracts and full texts of the publications. During each step, every publication was screened by at least two independent reviewers. Disagreements on study selection were settled by consensus and discussion with other reviewers if needed. The screening sheets applied by all reviewers are provided as part of the scoping review protocol registration [24]. Systematic reviews that were identified during the screening process were reviewed for any eligible references.

### Data extraction

A customized data extraction form was developed based on the JBI template for data extraction [26], using Microsoft Excel [Version 2503; Microsoft Corporation; Redmond, WA, USA]. Guided by published recommendations for HEEs [3, 20, 27] the form captured the following aspects: publication characteristics (year, country, funding source), health topic and type of intervention, prevention level (primary, secondary), study design (trial-based or model-based), population characteristics, type of economic evaluation (e.g. CEA, CUA, CBA), analytic perspective, time horizon, cost categories included, outcome measures, discount rates, reporting of ICERs and thresholds, modeling approach and assumptions, data sources, handling of uncertainty (e.g. sensitivity analyses), and reporting of supplementary analyses such as budget impact or return-on-investment calculations. Where necessary, additional variables were inductively added during pilot extraction to reflect reporting practices observed in the included studies. For data extraction, three reviewers (EJB, RMSV, MK) independently charted data from all included publications. A pilot extraction was performed, and regular team discussions were held to ensure the consistency and accuracy of the extraction. After finalization, the detailed extractions (Additional file 3) were summarized and transferred to categories by the first author. It should be noted that the categorization followed definitions established in the literature [3, 20, 27] and may not always match the reported classifications in the eligible studies (Additional file 4). The authors classified HEEs according to the evaluated endpoint [20]. Notably, in a German-speaking context, the term “cost-effectiveness analysis” is used more narrowly than in Anglo-American literature, where it often serves as an umbrella term encompassing both cost-effectiveness and cost utility analyses [3, 27, 28].

## Results

### Search results

**Fig. 1:**
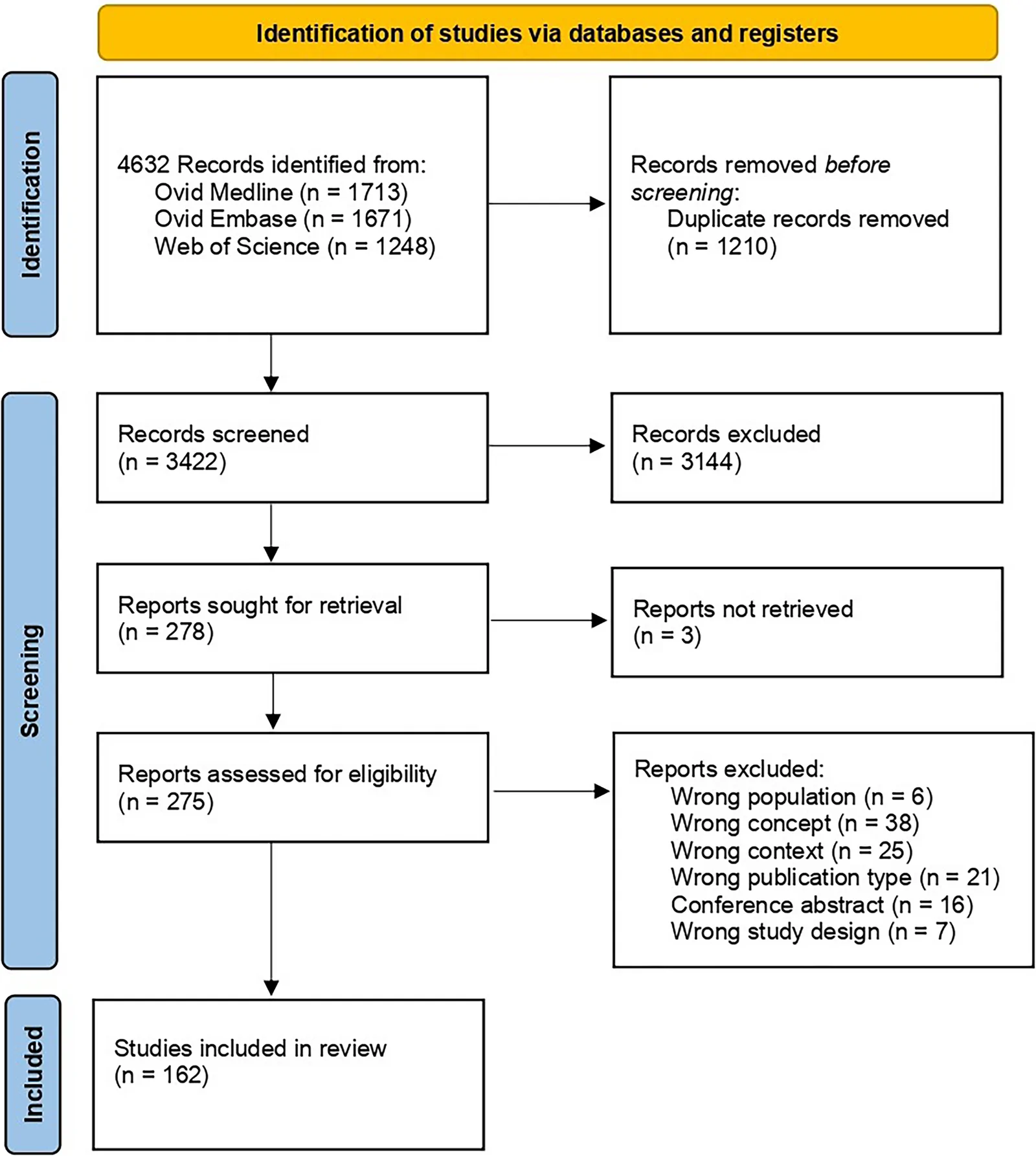
PRISMA-ScR flow chart of the studies identified, screened and included. Adapted from [29]

In total, 4,632 records were identified through the database searches. After removing 1,210 duplicates, 3,422 records were screened, of which 275 were assessed for eligibility. Ultimately, 162 primary research articles were included (Fig. 1).

### Content characteristics of eligible evidence

**Fig. 2:**
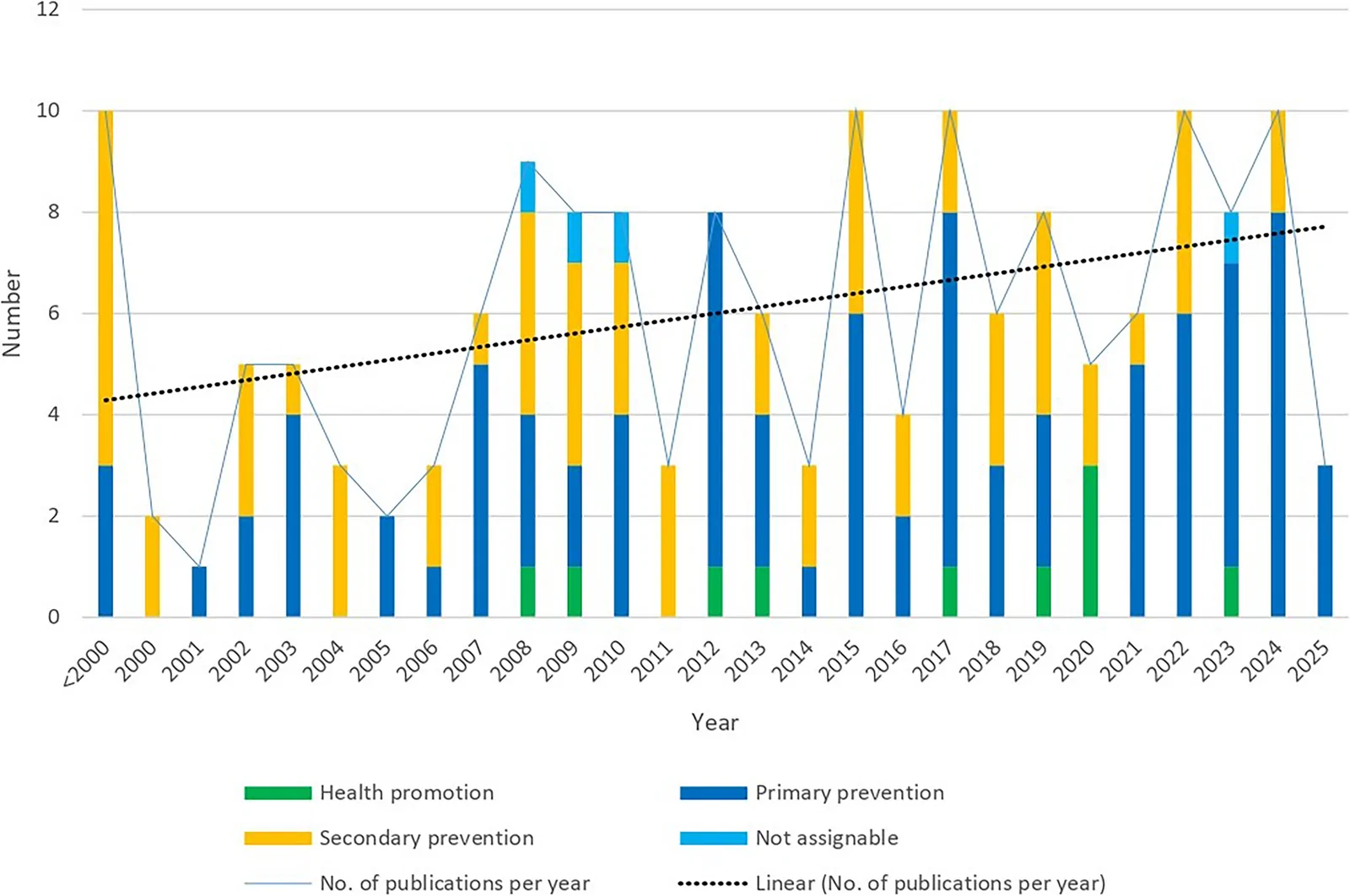
Number of publications by year and context with time trend

The included studies were published between 1994 and 2025, with peaks in 2015, 2017, 2022 and 2024 (6.2%; *n* = 10 in each year). In total, the volume of conducted HEEs increased over time (Fig. 2). The cost-effectiveness of interventions for health promotion was analyzed in 6.2% (*n* = 10) of the studies [30–39]. Primary preventive interventions were targeted in 53.7% (*n* = 87) of the studies [40–126]. Interventions for secondary prevention were researched in 37.7% (*n* = 61) [127–187]. In 2.5% (*n* = 4), although the studies met the eligibility criteria for HEEs, a clear assignment to primary or secondary prevention was not possible due to ambiguous or insufficient reporting of the intervention context [188–191].

In the 162 papers, 12 health topics were identified: dental health, environmental prevention, fall prevention, infection prevention, lifestyle programs, mental health, nutrition, occupational prevention, screening, smoking cessation, surgical prevention such as opportunistic salpingectomy performed to reduce ovarian cancer risk and vaccination. Screening interventions were the object of 34% (*n* = 55) of the analyzed studies, followed by interventions for vaccination (31.5%; *n* = 51) and lifestyle programs (12.4%; *n* = 20). In the years before 2000, only 10 publications (6.2%) were found, beginning in the year 1994. These HEEs conducted research on screening and vaccination interventions [45, 118, 121, 130, 134, 162, 167, 178, 181, 184] (Table 3). The most frequently thematized medical matters were vaccinations against pneumococcal diseases (8%; *n* = 13) [50, 51, 59, 70, 74, 82–85, 88, 117, 119, 120], followed by screening activities against colorectal cancer (7.4%; *n* = 12) [144, 145, 159, 161–163, 175, 177–179, 183, 185]. Third most, were studies focusing on screening and surgical interventions against breast, cervical or ovarian cancer (6.8%; *n* = 11) [66, 77, 95, 107, 128, 129, 134, 169, 170, 172, 184]. The research to pneumococcal diseases shows a focus in the year 2012 [74, 84, 117] and again in 2024 [70, 119]. Colorectal cancer is presented continuously over the years 1996 to 2024. Interventions against breast, ovarian or cervical cancer accumulate from the year 2015 onwards, with a peak in 2019 [95, 129, 169].

**Table 3:** Included studies by context and health topic (*n* = 162)

Context	Proportion (%)	Health topic	Proportion (%)	References^a^
Health promotion	6.2	Environmental prevention	1.2	Emmert-Fees et al., 2023 [30]; Leao et al., 2020 [35]
		Lifestyle program	3.1	Hoeflmayr & Hanewinkel, 2008 [31]; Kesztyus et al., 2013 [33]; Kesztyus et al., 2017 [32]; Lauer et al., 2020 [34]; Lier et al., 2020 [36]
		Mental health	0.6	Müller, G. et al., 2019 [37]
		Smoking cessation	1.2	Salize et al., 2009 [38]; Sargent et al., 2012 [39]
Primary prevention	53.7	Dental health	3.7	Jevdjevic et al., 2021 [73]; Schwendicke et al., 2017 [111]; Schwendicke & Bombeck, 2023 [109]; Schwendicke & Stolpe, 2017 [110]; Splieth & Flessa, 2008 [115]; Zimmer et al., 2018 [126]
		Environmental prevention	0.6	Sandmann et al., 2017 [102]
		Fall prevention	2.5	Gottschalk et al., 2022 [65]; Konnopka et al., 2023 [80]; Müller D. et al., 2015 [94]; Scheckel et al., 2021 [104]
		Infection prevention	1.9	Bartenschlager et al., 2022 [44]; Ellmann et al., 2022 [58]; Gandjour, 2023 [63]
		Lifestyle program	6.8	Brettschneider et al., 2015 [46]; Dams et al., 2024 [56]; Freund et al., 2024 [61]; Fuller et al., 2013 [62]; Häußler & Breyer, 2016 [68]; Huber et al., 2018 [71]; Icks et al., 2007 [72]; Kemmler et al., 2010 [78]; Meyer et al., 2005 [91]; Ogurtsova et al., 2024 [98]; Schuetz et al., 2013 [108]
		Nutrition	1.9	Mertens et al., 2012 [90]; Niedermaier et al., 2021 [97]; Sonntag et al., 2019 [114]
		Occupational prevention	1.9	Gaertner et al., 2007 [64]; Roesner et al., 2024 [100]; Strametz et al., 2025 [116]
		Surgical prevention	3.1	Hallsson et al., 2023 [66]; Kather et al., 2025 [77]; Müller D. et al., 2019 [95]; Saunders et al., 2023 [103]; Schrauder et al., 2017 [107]
		Vaccination	31.5	Aballéa et al., 2007 [40]; Aidelsburger et al., 2014 [41]; Averin et al., 2025 [42]; Banz et al., 2003 [43]; Beutels et al., 1996 [45]; Cai et al., 2021 [47]; Christensen et al., 2016 [48]; Cicchetti et al., 2010 [49]; Claes & Von der Schulenburg, 2003 [50]; Claes et al., 2009 [51]; Coudeville et al., 2005 [52]; Curran et al., 2021 [53]; Damm et al., 2015 [54]; Damm et al., 2017 [55]; Diel et al., 2001 [57]; Evers et al., 2007 [59]; Fischinger et al., 2010 [60]; Hammerschmidt et al., 2007 [67]; Hillemanns et al., 2009 [69]; Huang et al., 2024 [70]; Jiang et al., 2012 [74]; Joshi et al., 2024 [75]; Karmann et al., 2015 [76]; Kohli et al., 2022 [79]; Kotsopoulos et al., 2015 [81]; Kuchenbe- cker et al., 2018 [82]; Kuhlmann et al., 2012 [84]; Kuhlmann et al., 2017 [83]; Kühne et al., 2023 [85]; Largeron et al., 2017 [86]; Lee et al., 2008 [87]; Lloyd et al., 2008 [88]; Lugner et al., 2012 [89]; Mittendorf et al., 2003 [92]; Molnar et al., 2022 [93]; Müller M. et al., 2024 [96]; Preaud et al., 2015 [99]; Rychlik et al., 2003 [101]; Schobert et al., 2012 [105]; Scholz et al., 2022 [106]; Scuffham et al., 2002 [112]; Soergel et al., 2012 [113]; Strutton et al., 2012 [117]; Szucs et al., 1998 [118]; Ta et al., 2024 [119]; Talbird et al., 2010 [120]; Tormans et al., 1998 [121]; Ultsch et al., 2013 [122]; Van Oorschot et al., 2019 [123]; Windorfer et al., 2006 [124]; Wutzler et al., 2002 [125]
Secondary prevention	37.7	Fall prevention	1.9	Gandjour & Weyler, 2008 [142]; Heinrich et al., 2013 [147]; Kunigkeit et al., 2018 [158]
		Lifestyle program	0.6	Aljutaili et al., 2014 [127]
		Occupational prevention	1.2	Batzdorfer et al., 2002 [132]; Schneider & Häck, 2011 [175]
		Screening	34	Armstrong & Guest, 2020 [128]; Arnold et al., 2019 [129]; Barry et al., 1998 [130]; Barth et al., 2018 [131]; Becker et al., 2015 [133]; Beemsterboer et al., 1994 [134]; Birkemeyer et al., 2020 [135]; Diel et al., 2006 [136]; Diel et al., 2009 [137]; Diller et al., 2008 [138]; Eklund et al., 2024 [139]; Frankenberger et al., 2022 [140]; Gandjour, 2022 [143]; Gandjour et al., 2023 [141]; Gan- sen et al., 2019 [144]; Hagen et al., 2008 [145]; Heinemann & Bohnert, 2000 [146]; Hofer et al., 2018 [148]; Hübner et al., 2015 [149]; Icks et al., 2004 [150]; Kairys et al., 2022 [151]; König et al., 2000 [152]; König et al., 2002a [153]; König & Barry, 2002b [154]; König & Barry, 2004 [155]; Krauth et al., 2019 [156]; Krensel et al., 2021 [157]; Ladabaum et al., 2014 [159]; Lange et al., 2016 [160]; Lwin et al., 2024 [161]; Manus et al., 1996 [162]; Menges et al., 2006 [163]; Mewes et al., 2022 [164]; Mueller & Gandjour, 2008 [165]; Mueller & Gandjour, 2009 [166]; Müller E. et al., 1999 [167]; Mutters et al., 2016 [168]; Neusser et al., 2019 [169]; Petry et al., 2017 [170]; Rasch & Perleth, 2011 [171]; Ruile et al., 2015 [172]; Schaufler, 2009 [173]; Schaufler & Wolff, 2010 [174]; Schulz et al., 2009 [176]; Severin et al., 2015 [177]; Sieg & Brenner, 2007 [179]; Sieg et al., 1998 [178]; Sroczynski et al., 2011 [180]; Szucs et al., 1996 [181]; Treskova et al., 2017 [182]; Tscheulin & Drevs, 2010 [183]; Warmerdam et al., 1997 [184]; Webendörfer et al., 2004 [185]; Weber et al., 2013 [186]; Wolf et al., 2010 [187]
Health promotion, primary and secondary prevention	1.9	Lifestyle program	1.9	Friedrichs et al., 2009 [188]; Stock et al., 2008 [190]; Stock et al., 2010 [189]
Primary and secondary prevention	0.6	Mental health	0.6	Waldmann et al., 2023 [191]

^a^Reference of the studies identified in this scoping review

Among the studies analyzed, 78.4% (*n* = 127) originated in Germany, but in 21.6% (*n* = 35), the cooperation or research was conducted in other countries (e.g., the UK, the US or the Netherlands) using data that originated from Germany, (Table 4). Funding was reported in 67.3% of the studies, originating from institutional (33.3%; *n* = 54) and industrial (32.1%; *n* = 52) grants.

**Table 4:** References by country of origin and funding sources

Category	Proportion (%)	References*
Country of origin		
Germany	78.4	[31–34, 36–39, 41, 42, 44, 46, 50, 51, 54, 56–58, 60, 63–69, 71, 72, 76–78, 80, 82–86, 90–92, 94–107, 109–111, 113–116, 118, 119, 121–127, 130–133, 135–138, 141–158, 160–163, 165–191]
Other	21.6	[30, 35, 40, 43, 45, 47–49, 52, 53, 55, 59, 61, 62, 70, 73–75, 79, 81, 87–89, 93, 108, 112, 117, 120, 128, 129, 134, 139, 140, 159, 164]
Funding		
Institutional	33.3	[30, 35, 40, 43, 45, 47–49, 52, 53, 55, 59, 61, 62, 70, 73–75, 79, 81, 87–89, 93, 108, 112, 117, 120, 128, 129, 134, 139, 140, 159, 164]
Industry	32.1	[42, 43, 50–54, 60, 62, 69, 74–76, 78, 79, 81, 82, 84–86, 92, 93, 96, 99, 101, 105, 106, 108, 112, 117, 119–121, 123, 124, 126, 128, 130, 131, 135, 137, 146, 149, 156, 159, 164, 170, 174, 175, 185–187]

*References of the studies identified in this review

### Methodological characteristics of eligible evidence

Among the reported outcomes, 32.1% (*n* = 52) of the identified types of HEEs represented a CEA, 13.6% (*n* = 22) a CUA and 12.4% (*n* = 20) a CBA (Table 5). The percentage identified as CCA was 4.9% (*n* = 8), and that identified as CMA was 4.3% (*n* = 7). BIA was conducted for 6.2% (*n* = 10) of all publications. Additionally, 26.6% (*n* = 43) reported more than one type of outcome and therefore could be classified as more than one type, led by the combination of CEA and CUA in 12.4% (*n* = 20) of the papers. A CEA was also the most frequently used base type (5 of 8 combinations).

**Table 5:** Included studies by type of HEE (*n* = 162)

Context	Proportion (%)	References^a^
Exclusive CEA	32.1	Aljutaili et al., 2014 [127]; Bartenschlager et al., 2022 [44]; Claes & Von der Schulenburg, 2003 [50]; Gandjour et al., 2003 [141]; Gandjour, 2023 [63]; Gansen et al., 2019 [144]; Gaertner et al., 2007 [64]; Hagen et al., 2008 [145]; Heinrich et al., 2013 [147]; Huang et al., 2024 [70]; Huber et al., 2018 [71]; Icks et al., 2004 [150]; Icks et al., 2007 [72]; Jiang et al., 2012 [74]; Kairys et al., 2022 [151]; Kather et al., 2025 [77]; Kesztyus et al., 2013 [33]; Kesztyus et al., 2017 [32]; Kohli et al., 2022 [79]; König et al., 2000 [152]; König et al., 2002 [153]; König & Barry, 2002 [154]; Konnopka et al., 2022 [80]; Krauth et al., 2019 [156]; Kuchenbecker et al., 2018 [82]; Kühne et al., 2023 [85]; Largeron et al., 2017 [86]; Leao et al., 2020 [35]; Lier et al., 2020 [36]; Lloyd et al., 2008 [88]; Lugner et al., 2012 [89]; Lwin et al., 2024 [161]; Mertens et al., 2012 [90]; Mewes et al., 2022 [164]; Meyer et al., 2005 [91]; Mittendorf et al., 2003 [92]; Müller G. et al., 2019 [37]; Müller M et al., 2024 [96]; Rychlik et al., 2003 [101]; Salize et al., 2009 [38]; Schulz et al., 2009 [176]; Schwendicke et al., 2017 [111]; Schwendicke & Bombeck, 2023 [109]; Schwendicke & Stolpe, 2017 [110]; Scuffham et al., 2002 [112]; Severin et al., 2015 [177]; Splieth & Flessa, 2008 [115]; Sroczynski et al., 2011 [180]; Szucs et al., 1996 [181]; Treskova et al., 2017 [182]; Warmerdam et al., 1997 [184]; Wutzler et al., 2002 [125]
Exclusive CUA	13.6	Averin et al., 2025 [42]; Brettschneider et al., 2015 [46]; Curran et al., 2021 [53]; Evers et al., 2007 [59]; Gottschalk et al., 2022 [65]; Hillemanns et al., 2009 [69]; Hofer et al., 2018 [148]; Jevdjevic et al., 2021 [73]; König & Barry, 2004 [155]; Kuhlmann & Von der Schulenburg, 2017 [83]; Lauer et al., 2020 [34]; Molnar et al., 2022 [93]; Müller D. et al., 2015 [94]; Ogurtsova et al., 2024 [98]; Schaufler & Wolff, 2010 [174]; Schobert et al., 2012 [105]; Scholz et al., 2022 [106]; Schuetz et al., 2013 [108]; Soergel et al., 2012 [113]; Sonntag et al., 2019 [114]; Talbird et al., 2010 [120]; Waldmann et al., 2023 [191]
Exclusive CBA	12.4	Barry et al., 1998 [130]; Batzdorfer et al., 2002 [132]; Becker et al., 2015 [133]; Diller et al., 2008 [138]; Ellmann et al., 2022 [58]; Frankenberger et al., 2022 [140]; Friedrichs et al., 2009 [188]; Hammerschmidt et al., 2007 [67]; Häußler & Breyer, 2016 [68]; Kotsopoulos et al., 2015 [81]; Lange et al., 2016 [160]; Menges et al., 2006 [163]; Müller E. et al., 1999 [167]; Niedermaier et al., 2021 [97]; Sandmann et al., 2015 [102]; Sargent et al., 2012 [39]; Schneider & Häck, 2011 [175]; Sieg & Brenner, 2007 [179]; Strametz et al., 2025 [116]; Windorfer et al., 2006 [124]
Exclusive CCA	4.9	Hübner et al., 2015 [149]; Kemmler et al., 2010 [78]; Krensel et al., 2021 [157]; Manus et al., 1996 [162]; Saunders et al., 2023 [103]; Sieg et al., 1998 [178]; Webendörfer et al., 2004 [185]; Weber et al., 2013 [186]
Exclusive CMA	4.3	Diel et al., 2006 [136]; Diel et al., 2009 [137]; Heinemann & Bohnert, 2000 [146]; Stock et al., 2008 [190]; Stock et al., 2010 [189]; Tscheulin & Drevs, 2010 [183]; Zimmer et al., 2018 [126]
Exclusive BIA	6.2	Barth et al., 2018 [131]; Cicchetti et al., 2010 [49]; Eklund et al., 2024 [139]; Karmann et al., 2015 [76]; Neusser et al., 2019 [169]; Petry et al., 2017 [170]; Rasch et al., 2011 [171]; Roesner et al., 2024 [100]; Ruile et al., 2015 [172]; Wolf et al., 2010 [187]
CEA, CUA	12.4	Aballéa et al., 2007 [40]; Aidelsburger et al., 2014 [41]; Arnold et al., 2019 [129]; Birkemeyer et al., 2020 [135]; Cai et al., 2021 [47]; Christensen et al., 2016 [48]; Damm et al., 2017 [55]; Fuller et al., 2013 [62]; Gandjour, 2022 [143]; Hallsson et al., 2023 [66]; Ladabaum et al., 2014 [159]; Lee et al., 2008 [87]; Müller D. et al., 2019 [95]; Preaud et al., 2015 [99]; Schaufler et al., 2009 [173]; Schrauder et al., 2017 [107]; Strutton et al., 2012 [117]; Ta et al., 2024 [119]; Ultsch et al., 2013 [122]; Van Oorschot et al., 2019 [123]
CEA, CBA	4.9	Banz et al., 2003 [43]; Beutels et al., 1996 [45]; Coudeville et al., 2005 [52]; Diel et al., 2001 [57]; Fischinger et al., 2010 [60]; Hoeflmayr & Hanewinkel, 2008 [31]; Mutters et al., 2016 [168]; Tormans et al., 1998 [121]
CEA, BIA	3.1	Dams et al., 2024 [56]; Kuhlmann et al., 2012 [84]; Kunigkeit et al., 2018 [158]; Scheckel et al., 2021 [104]; Szucs et al., 1998 [118]
CEA, CUA, CBA	2.5	Claes et al., 2009 [51]; Damm et al., 2015 [54]; Beemsterboer et al., 1994 [134]; Freund et al., 2024 [61]
CEA, CUA, BIA	0.6	Emmert-Fees et al., 2023 [30]
CUA, BIA	1.9	Gandjour & Weyler, 2008 [142]; Mueller D. & Gandjour, 2008 [165]; Müller D. & Gandjour, 2009 [166]
CUA, CCA	0.6	Joshi et al., 2023 [75]
CBA, CMA	0.6	Armstrong & Guest, 2020 [128]

*CEA* Cost-effectiveness analysis,* CUA *Cost-utility analysis, *CBA* Cost-benefit analysis, *CCA* Cost-Consequence analysis, *CMA* Cost-minimization analysis,* BIA *Budget impact analysis, *HEE* Health economic evaluation

^a^Reference of the studies identified in this scoping review

The population size in the analyzed studies ranged from 1 person to approximately 83 Mio people (entire German population), with the median being 90,353 persons. In 24.1% (*n* = 39) of the studies, no population size was reported (Table 6). In 93.8% (*n* = 152) of all HEEs analyzed, a stand-alone economic study was presented, whereas 6.2% (*n* = 10) carried out an economic analysis in addition to a clinical effectiveness study (Table 6).

**Table 6:** Summary of methodological characteristics (*n* = 162)

Category	Results (*n* and %)	References^a^
Population size		
Not reported	39 (24.1)	[30, 35, 45, 51, 54, 57–59, 66, 67, 69, 75, 79, 82, 83, 86, 94, 96–98, 101–104, 106, 118, 124, 125, 128, 134, 139–141, 143, 149, 158, 171, 180, 184]
Economic evaluation design		
Stand-alone economic study	152 (93.8)	[ 30-38, 40-77, 79, 80-109, 111-159, 161-175, 177-181, 183, 187, 189-191]
On top of a clinical effectiveness study	10 (6.2)	[39, 61, 78, 110, 160, 176, 182, 185, 186, 188]
Applied perspective		
Third-party payer (only)	85 (52.5)	[35, 38, 39, 51, 56, 60, 66, 68, 69, 71, 74, 76–78, 80, 81, 85, 86, 88, 89, 91, 92, 94–96, 101, 103–105, 107–112, 115, 117, 118, 120, 121, 126–129, 131–133, 135, 136, 138, 139, 144–148, 151–155, 158, 159, 161, 163, 165–167, 169–172, 174,177–182, 184–186, 188–190]
Societal (only)	23 (14.2)	[31–33, 42, 46, 49, 57, 58, 63, 64, 70, 73, 79, 87, 102, 106, 119, 123, 130, 137, 156, 157, 191]
Healthcare provider (only)	5 (3.1)	[100, 116, 149, 162, 168]
Patient (only)	3 (1.9)	[34, 140, 143]
Employer (only)	1 (0.6)	[124]
Third-party payer and societal (combination)	34 (21)	[30, 36, 37, 40, 41, 43, 45, 47, 48, 50, 52, 54, 55, 62, 65, 67, 72, 75, 82–84, 90, 93, 98, 99, 122, 125, 141, 142, 150, 160, 164, 183, 187]
Societal (total)	58 (35.8)	[30–33, 36, 37, 40–43, 45–50, 52, 54, 55, 57, 58, 61–65, 67, 70, 72, 73, 75, 79, 82–84, 87, 90, 93, 98, 99, 102, 106, 119, 122, 123, 125, 130, 137, 141, 142, 150, 156, 157, 160, 164, 183, 187, 191]
Not reported	8 (4.9)	[44, 53, 59, 97, 113, 114, 134, 176]
Considered cost types		
Direct medical costs (total)	155 (95.7)	[30, 31, 36–99, 101–115, 117–131, 133–191]
Direct medical costs (only)	75 (48.4)	[38, 39, 46, 50, 56, 59, 60, 62, 66, 68, 69, 74, 77, 78, 80, 86, 91, 92, 94–97, 101, 104, 105, 107, 108, 110, 112, 113, 115, 117, 120, 126, 127, 129, 131, 133–136, 138, 139, 144–147, 149, 151, 154, 155, 157–159, 162, 164, 167–174, 176, 177, 179–182, 184, 188–191]
Indirect costs	65 (40.1)	[30, 31, 37, 40–45, 47–49, 51–55, 57, 58, 61, 63–65, 67, 70, 72, 73, 75, 76, 79, 81–85, 88–90, 93, 98, 99, 106, 114, 118, 119, 121–125, 128, 130, 132, 137, 141, 150, 156, 160, 175, 178, 183, 185–187]
Private payments	14 (8.6)	[34, 40, 73, 83, 84, 89, 90, 93, 109, 111, 119, 123, 143, 160]
Applied discount rate on costs		
Rate applied	109 (67.3)	[30, 31, 35–38, 40–43, 45–48, 50–55, 57, 59, 60, 63–66, 68, 69, 71, 73, 75, 77, 79, 81, 83, 85–90, 92–96, 98, 99, 104–106, 108, 109, 111–115, 118–123, 125–131, 134–137, 139, 141, 142, 144, 145, 147, 148, 150, 151, 155, 156, 158, 159, 161, 163, 165–167, 169, 170, 171, 173–175, 177, 179–184, 187, 191]
None rate applied	49 (30.3)	[32–34, 39, 44, 49, 56, 58, 62, 67, 70, 72, 74, 76, 78, 80, 82, 84, 91, 97, 100–103, 107, 116, 124, 132, 133, 140, 143, 146, 149, 152–154, 157, 160, 162, 164, 168, 172, 176, 178, 181, 185, 186, 189, 190]
Not applicable	3 (1.9)	[138, 61, 188]
Reported outcomes		
Clinical endpoints (only)	55 (34)	[31, 32, 36–38, 44, 56–58, 60, 64, 68, 72, 76, 78, 80, 90, 91, 102–104, 109, 110, 115, 116, 118, 121, 122, 126, 128, 130, 131, 137–139, 141, 143, 146, 147, 150, 152–154, 160, 162–164, 167, 168, 170–172, 183, 185, 186]
QALYs gained	60 (37)	[30, 40–42, 46–48, 51, 53–55, 59, 61, 62, 65, 66, 69–71, 75, 77, 79, 83, 85–87, 89, 93–96, 98, 99, 105, 106, 108, 113, 114, 117, 119, 120, 122, 123, 129, 134, 135, 142, 143, 148, 151, 155, 156, 158, 159, 161, 165, 166, 173, 174, 191]
LYG	36 (22.2)	[30, 40, 42, 43, 51, 52, 55, 63, 66, 69, 70, 77, 82–85, 88, 92, 95, 98 , 101, 105, 107, 112, 117, 120, 125, 127, 128, 134, 144, 145, 177, 180, 182, 184]
DALY	2 (1.2)	[73, 111]
LYS	6 (3.7)	[45, 48, 50, 113, 148, 181]
YLL	3 (1.9)	[92, 97, 117]
HLY	1 (0.6)	[35]
QALYs lost	2 (1.2)	[117, 156]
Reported ICER		
Reporting of an ICER	104 (64.2)	[30–32, 37, 38, 40–43, 45–48, 50, 51, 53–57, 59–66, 69–72, 76, 77, 79, 80, 82–87, 89–95, 97–99, 104–115, 117–119, 121–123, 125, 127–129, 134, 135, 141–145, 147, 148, 150, 151, 153–156, 158, 159, 161, 164–166, 171, 173, 174, 177, 180–184]
Reporting of more than 1 ICER	30 (18.5)	[41–43, 45, 48, 51, 55, 56, 61, 66, 70, 77, 83, 85, 87, 95, 99, 112, 113, 117, 119, 122, 123, 125, 128, 134, 143, 148, 164, 182]
Proportion of all reported ICER Incremental costs per incremental …		
QALY gained	56 (42.4)	[30, 40–42, 46–48, 51, 53–55, 59, 61, 62, 65, 66, 69–71, 77, 79, 83, 85–87, 89, 93–95, 98, 99, 105, 106, 108, 113, 114, 117, 119, 122, 129, 134, 135, 142, 143, 148, 151, 155, 156, 158, 159, 161, 165, 166, 173, 174, 191]
Clinical endpoint	43 (32.6)	[31, 32, 37, 38, 41, 43, 45, 48, 51, 56, 57, 60, 61, 64, 72, 76, 80, 87, 90, 91, 99, 104, 109, 110, 112, 115, 118, 119, 121–123, 125, 128, 141, 143, 147, 150, 153, 154, 164, 171, 182, 183]
LYG	28 (21.2)	[42, 43, 51, 52, 55, 63, 66, 70, 77, 82–85, 92, 95, 101, 107, 112, 117, 125, 127, 134, 144, 145, 177, 180, 182, 184]
Other outcomes	7 (5.3)	[45, 50, 97, 111, 113, 148, 181]
Data references shown in table form		
Yes	82 (50.6)	[30, 35, 40, 41, 43, 46, 48, 49, 51, 54–56, 58, 59, 62–64, 66, 69, 72, 74–76, 81–89, 91, 92, 94–97, 99, 103–106, 108–114, 117, 119, 120, 122, 124, 128, 129, 131, 135–137, 139–144, 148–151, 155, 158, 161, 164–166, 169, 170, 173, 180, 187]
No	61 (37.7)	[31, 33, 38, 39, 42, 44, 45, 47, 50, 52, 53, 57, 60, 61, 65, 67, 68, 70, 71, 73, 77–80, 93, 98, 100–102, 115, 116, 118, 121, 123, 125–127, 130, 132, 134, 138, 145, 147, 152–154, 156, 157, 159, 163, 167, 171, 172, 174, 177, 179, 181–184, 191]
Not applicable	19 (11.7)	[32, 34, 36, 37, 90, 107, 133, 146, 160, 162, 168, 175, 176, 178, 185, 186, 188–190]
Reported cost-effectiveness of intervention		
Yes, cost-effective	138 (85.2)	[30–33, 35–40, 42–45, 47, 49–55, 58–63, 66–69, 71, 73, 75–77, 79, 81–103, 105–108, 110–121, 123–130, 132, 134–140, 142, 144, 146–182, 184, 185, 187–190]
No, not cost-effective	20 (12.4)	[41, 46, 48, 56, 57, 64, 65, 70, 72, 74, 80, 104, 109, 122, 133, 143, 145, 183, 186, 191]
No clear statement	4 (2.5)	[34, 78, 131, 141]
Application of a cost-effectiveness threshold		
No	114 (70.4)	[30–34, 36–39, 41, 43–45, 49–52, 56–60, 64, 65, 67–69, 72–74, 76–79, 81, 84–86, 88, 90–92, 94, 99–103, 107, 108, 110, 112, 113, 115, 116, 118–121, 124–126, 128, 130–141, 143–146, 148–155, 157, 160, 162, 163, 165, 167–176, 178–183, 185–190]
Yes	48 (29.6)	[35, 40, 42, 46–48, 53–55, 61–63, 66, 70, 71, 75, 80, 82, 83, 87, 89, 93, 95–98, 104–106, 109, 111, 114, 117, 122, 123, 127, 129, 142, 147, 156, 158, 159, 161, 164, 166, 177, 184, 191]
50,000 € per QALY gained [Modus]	19 (11.7)	[40, 42, 46, 53, 55, 62, 70, 75, 82, 93, 98, 117, 122, 123, 127, 147, 158, 159, 177]

*CEA* Cost-effectiveness analysis, *CUA* Cost-utility analysis, *CBA *Cost-benefit analysis,* CCA *Cost-Consequence analysis, *CMA* Cost-minimization analysis, *BIA* Budget impact analysis,* HEE *Health economic evaluation

^a^Reference of the studies identified in this scoping review

Across the included studies, the most common analytical perspective was that of a third-party payer, typically a social health insurance, which was used in 52.5% (*n* = 85) of all studies (Table 6). A solely societal perspective was applied in 14.2% (*n* = 23) of the studies, whereas 3.1% (*n* = 5) evaluated interventions from the healthcare provider perspective (e.g., hospitals). Only 1.9% (*n* = 3) adopted a patient perspective, and 0.6% (*n* = 1) took an employer perspective. Multiple perspectives were used in 22.8% (*n* = 37) of the studies. Most frequently, the third-party payer and societal perspectives were both considered in the analysis with results typically reported separately (21%; *n* = 34). Overall, 35.8% (*n* = 58) of all studies included a societal viewpoint, either alone or alongside others. For 4.9% (*n* = 8) of all HEEs, the applied perspective was not reported.

The considered cost types comprised direct medical costs, such as intervention or treatment costs, in 95.7% (*n* = 155) of the evaluations (Table 6). In 51.6% (*n* = 80) of the HEEs, a combination with other cost types was applied. Indirect costs, describing productivity losses due to disease or disability, were co-considered in 40.1% (*n* = 65) of all studies, with private payments of patients accounting for 8.6% (*n* = 14). The applied discount rate on costs ranged between 0% and 5%, with the median located at 3%. For 30.3% (*n* = 49) of the HEEs, no discount rate on costs was applied.

Clinical endpoints, such as cases avoided or deaths prevented, were reported exclusively in 34% (*n* = 55) of the studies (Table 6). In addition to clinical endpoints, the most commonly reported outcome was the QALY (37%; *n* = 60), followed by life-years gained (LYG) (22.2%; *n* = 36). Other measures of life expectancy or health-related quality of life, including life years saved (LYS), years of life lost (YLL), and disability-adjusted life years (DALYs), were identified in 8.6% of the studies (*n* = 14).

The reporting of an ICER was observed in 64.2% (*n* = 104) of all the evaluations analyzed (Table 6). In 18.5% (*n* = 30) of these studies, more than one ICER was presented. Among all the reported ICERs (*n* = 132), the representation consisted of incremental costs per (i) incremental QALY gained (42.4%; *n* = 56), (ii) incremental clinical endpoint (32.6%; *n* = 43), (iii) incremental LYG (21.2%; *n* = 28) and (iv) incremental other outcomes such as LYS, YLL, DALY or QALY lost (8%; *n* = 13). Additionally, 4.3% (*n* = 7) of all HEEs reported a Return on Investment (ROI) calculation [36, 54, 71, 75, 112, 124, 128].

Information on the data sources used in the HEEs was presented in tabular form in 50.6% (*n* = 82) of the studies (Table 6). For 37.7% (*n* = 61) of the studies, information on data sources was provided but not in table form. For 11.7% (*n* = 19) of the studies, this reporting element was not applicable because the studies relied on real-world data. 

### Cost-effectiveness of eligible studies

Among the included HEEs, 85.2% (*n* = 138) reported, according to the authors’ own conclusions, that the evaluated intervention was cost‑effective or cost‑saving in at least one analysis (base case or scenario analysis) (Table 6). In contrast, 12.4% (*n* = 20) concluded that the intervention was not cost-effective. Clear statements regarding cost-effectiveness were missing or not applicable in 2.5% of the studies (*n* = 4). This classification was based on the primary cost‑effectiveness statements reported by study authors and does not distinguish between base‑case results and alternative scenario findings. It should be noted that authors frequently described interventions as cost‑effective even in the absence of an explicitly reported cost‑effectiveness threshold, for example based on dominance, comparison with results from previous studies, or qualitative interpretation of incremental costs and outcomes.

Overall, 29.6% (*n* = 48) of the 162 included studies applied a cost-effectiveness threshold (Table 6). Besides other thresholds (e.g., cost per LYG), reported values ranged from 0 to 500,000 Euro per QALY gained, with a median of 30,000 Euro per QALY gained. The most frequently used threshold was 50,000 Euro per QALY gained, applied in 11.7% (*n* = 19) of all studies.

### Characteristics of modeling studies

Among the included studies, 75.9% (*n* = 123) used a model-based approach to simulate costs and outcomes (Table 7). Among these model-based studies, 44.7% (*n* = 55) applied Markov models. Decision trees were used in 16.3% (*n* = 20), and 7.3% (*n* = 9) employed a combination of Markov models and decision trees. Microsimulation models were used in 8.9% (*n* = 11), and other model types in 11.4% (*n* = 14). In an additional 11.4% (*n* = 14), the model type was not further specified.

**Table 7:** Summary of modeling study details (*N* = 123)

	Result (*n* and %)	Reference^a^	
Model-based approaches			
Modeling studies	123 (75.9)	[30, 35, 36, 40–45, 47–60, 63, 66–75, 77, 79, 81–96, 98–106, 108–115, 117–126, 128–131, 134–137, 139–145, 148–153, 155, 156, 158, 159, 161, 163–167, 169–174, 177, 179–182, 184, 187]	
Type of model			
Markov model	55 (44.7)	[35, 41–43, 45, 48, 50–53, 56, 59, 63, 66, 68–71, 74, 82–85, 87, 92, 94–96, 98, 100, 104, 109, 113, 117–119, 121–123, 126, 129, 131, 135, 140, 142, 145, 148, 151, 156, 158, 159, 165, 166, 169, 180]	
Decision tree	20 (16.3)	[40, 47, 72, 90, 91, 112, 124, 136, 137, 141, 149, 150, 152, 153, 163, 164, 167, 171, 181, 187]	
Decision tree and Markov model in combination	9 (7.3)	[75, 99, 103, 128, 139, 144, 155, 170, 177]	
Microsimulations	11 (8.9)	[30, 44, 73, 110, 111, 114, 134, 173, 174, 182, 184]	
Others	14 (11.4)	[49, 55, 67, 79, 86, 89, 93, 101, 105, 106, 108, 115, 161, 172]	
Not specified	14 (11.4)	[36, 54, 57, 58, 60, 77, 81, 88, 102, 120, 125, 130, 143, 179]	
Applied time horizons			
Lifetime horizon	34 (27.6)	[35, 42, 53, 63, 66, 71, 81, 82, 85, 87, 95, 98, 99, 105, 108–111, 114, 115, 122, 123, 126, 129, 135, 151, 159, 161, 165, 166, 174, 177, 180, 182]	
1 Year	12 (9.8)	[40, 59, 75, 89, 100, 103, 117, 120, 141, 143, 150, 164]	
10 Years	11 (8.9)	[36, 50, 54, 70, 73, 119, 120, 163, 169, 170, 179]	
Not reported	6 (4.9)	[77, 88, 101, 149, 173, 181]	
Sensitivity Analyses			
Univariate DSA	102 (87.2)	[35, 40–45, 47, 50–57, 59, 60, 63, 66–75, 77, 79, 81–92, 95, 96, 98, 99, 102–106, 108–112, 114, 115, 118, 119, 121–123, 125, 128, 129, 131, 134–137, 139–143, 145, 148–150, 152, 154–156, 158, 159, 164–167, 170–172, 174, 177, 179–182, 184, 187]	
Multivariate DSA	18 (15.4)	[52, 54–56, 59, 84, 91, 94, 122, 124, 136, 141, 142, 150, 152, 169, 180, 187]	
PSA	55 (47)	[30, 40, 42, 43, 47–49, 53, 54, 56, 58, 68, 70–73, 75, 77, 79, 82–85, 93–95, 98–100, 103, 104, 106, 109–111, 119, 122, 123, 128, 129, 135, 137, 141, 142, 148, 150, 151, 155, 158, 159, 161, 164–166, 177]	
Scenario Analyses	34 (29)	[30, 42, 47, 48, 50, 69, 70, 74, 75, 77, 79, 81, 82, 85, 86, 93, 94, 96, 103, 104, 109, 114, 117, 119, 120, 122, 123, 125, 141, 144, 152, 156, 177, 180]	
Threshold Analyses	9 (7.7)	[41, 45, 82, 83, 109, 111, 121, 123, 159]	
NMB / CEAC	15 (12.8)	[71, 82, 94, 98, 99, 104, 109, 114, 129, 142, 161, 164–166, 177]	
VOI	3 (2.6)	[110, 111, 177]	
Application of			
one type of SA	46 (37.4)	[35, 44, 49, 51, 57, 58, 60, 66, 67, 87–90, 92, 100, 102, 105, 108, 112, 115, 117, 118, 120, 124, 125, 131, 134, 139, 140, 143–145, 148, 149, 151, 154, 167, 169–172, 174, 179, 181, 182, 184]	
two types of SA	31 (25.2)	[30, 40, 41, 43, 45, 48, 50, 52, 53, 55, 59, 63, 68, 69, 72, 73, 81, 86, 91, 93, 95, 96, 106, 121, 128, 135–137, 155, 156, 187]	
three types of SA	24 (19.5)	[42, 47, 54, 56, 70, 74, 75, 77, 79, 83–85, 99, 103, 119, 122, 141, 150, 152, 158, 159, 161, 164, 180]	
four types of SA	10 (8.1)	[71, 98, 104, 110, 114, 123, 129, 142, 165, 166]	
more than four types of SA	5 (4)	[82, 94, 109, 111, 177]	
Applied cycle length			
Modus: 1 year	37 (57.8)	[43, 50, 51, 53, 56, 63, 66, 68, 70, 71, 74, 75, 82–84, 87, 92, 94–96, 98, 109, 119, 123, 126, 128, 129, 139, 144, 145, 155, 159, 165, 166, 169, 177, 180]	
Data origin			
Non-German	100 (81.3)	[30, 35, 36, 40–45, 47–52, 54, 55, 57, 63, 66, 69, 70, 73, 75, 77, 81–89, 92, 94–96, 98–106, 108–115, 117, 119–126, 128, 129, 131, 134, 135, 137, 139–144, 148–152, 155, 156, 159, 161, 165, 166, 169–174, 177, 179–182, 184, 187)	
German	22 (17.9)	[53, 56, 58–60, 67, 68, 72, 74, 79, 90, 91, 93, 118, 130, 136, 145, 154, 158, 163, 164, 167]	
Unclear	1 (0.8)	[71]	
Report on lack of German data in study limitations			
Yes	69 (56.1)	[30, 35, 40–42, 44, 45, 47, 48, 50, 51, 54, 56, 60, 66, 67, 69, 74, 79, 82–87, 89, 92, 93, 95, 96, 98, 99, 105, 106, 108, 110–114, 117, 119, 120, 122, 125, 126, 129, 134, 135, 137, 139, 141, 142, 148, 149, 151, 152, 155, 156, 158, 159, 161, 164–166, 169, 180, 182, 184]	
No	51 (41.5)	[36, 43, 49, 52, 53, 55, 57, 58, 59, 63, 68, 70–73, 75, 77, 81, 88, 90, 91, 94, 100–104, 109, 115, 118, 123, 124, 128, 130, 131, 136, 140, 143, 145, 150, 154, 163, 167, 170–172, 174, 177, 179, 181, 187]	
Not reported	3 (2.4)	[121, 144, 173]	

*DSA* Deterministic sensitivity analysis, *PSA* Probabilistic sensitivity analysis, *NMB* Net monetary benefit, *CEAC* Cost-effectiveness acceptability curve, *VOI* Value of information, *SA* Sensitivity analysis

^a^Reference of the studies identified in this scoping review

The model studies simulated a median time horizon of 20 years, ranging from 6 weeks to 120 years. A lifetime horizon was applied in 27.6% (*n* = 34), whereas 4.9% (*n* = 6) did not report the time horizon. The most frequently used fixed time horizons were 1 year (9.8%; *n* = 12) and 10 years (8.9%; *n* = 11).

Sensitivity analyses (SAs) were conducted for 95.1% (*n* = 117) of all model-based HEEs. Of these, 37.4% (*n* = 46) conducted one type of SA, 25.2% (*n* = 31) used two types, 19.5% (*n* = 24) used three types, 8.1% (*n* = 10) used four types, and 4% (*n* = 5) applied more than four types. The most commonly reported SAs were univariate deterministic sensitivity analyses (DSA; 87.2%; *n* = 102), probabilistic sensitivity analyses (PSA; 47%; *n* = 55), and scenario analyses (29%; *n* = 34). Less frequently reported were multivariate DSAs (15.4%; *n* = 18), threshold analyses (7.7%; *n* = 9), cost-effectiveness acceptability curves (CEACs; 12.8%; *n* = 15), and value-of-information analyses (VOIs; 2.6%; *n* = 3).

Among the models that use discrete-time simulations, the cycle lengths ranged from 1 day to 10 years, with a median of 150 days. The most frequently applied cycle length was 1 year per Markov cycle (57.8%; *n* = 37) of all Markov cycle lengths reported (*n* = 64).

Data from non-German sources were utilized in 81.3% (*n* = 100) of all model-based HEEs. In 0.8% (*n* = 1), the origin of the data used in the model was unclear. Correspondingly, 56.1% (*n* = 69) of the modeling studies reported a lack of German data in their study limitations. In 2.4% (*n* = 3), no study limitations were reported. 

## Discussion

This scoping review provides an overview of HEEs in the context of health promotion as well as primary and secondary prevention in Germany, with the aim of supporting future research and informing evidence-based decision-making. A total of 162 eligible studies published from 1994 onward were identified.

### Content characteristics

Most HEEs focused on primary preventive interventions and covered 12 distinct health topics. As expected, a substantial proportion of the studies evaluated screening and vaccination programs. Among these, particular emphasis has been placed on vaccinations against pneumococcal disease and on screening or surgical interventions aimed at preventing colorectal, breast, ovarian, and cervical cancer. Given the substantial clinical and economic burden associated with cancer, this concentration is expected. Lifestyle programs constitute the third most common category. These studies addressed a range of behavioral interventions targeting individuals at risk and aimed to promote healthier lifestyles. However, they accounted for only 12.4% of all included HEEs. This relatively small share may indicate the limited implementation of health-promoting lifestyle programs in Germany. It may likewise reflect the absence of accompanying economic evaluations rather than a limited availability of such programs.

In contrast to a previous scoping review on methodological issues in HEEs [21], fewer CEAs and CUAs were identified (previous review vs. this review: CEAs 42% vs. 32%; CUAs 20% vs. 14%), but more cost-benefit analyses (CBAs: 6% vs. 12%) and more reports of return on investment (ROI: 0.4% vs. 4.3%) were reported. Our review also included CMAs and BIAs, which may partly explain these differences. The role of CMAs as a form of full economic evaluation continues to be debated in the literature [3, 192]. Compared with the previous review, we found a substantially greater share of modeling studies (44% vs. 76%) and a greater consideration of indirect costs (23% vs. 40.1%). In contrast, the societal perspective was less frequently adopted in our sample (46% vs. 35.8%), a finding that stands in contrast to the recommendations of the Hanover Consensus [20]. Overall, the increased inclusion of indirect costs suggests a positive trend toward broader cost measurement in HEEs, even if the choice of analytic perspective remains heterogeneous.

### Heterogeneity of publications

The studies analyzed in our review revealed an expected highly heterogeneous landscape of HEEs for interventions related to health promotion and disease prevention. This heterogeneity was combined with often challenging reporting for external readers. Structural differences, the use of various vocabularies and the inflationary use of the term “cost-effectiveness” are some of the observed obstacles and may lead to limitations in value for decision makers [21]. Additionally, deviations from well-established general standards of economic evaluation, such as CHEERS [13], were observed, although the extent and implications of these deviations varied across studies. As the main aim of HEEs is to ensure the ability of interpretation and usefulness for decision making, heterogeneous reporting is a problem that consortiums such as ISPOR or the Hanover Consensus have intended to improve. Nevertheless, even in earlier years, no consistent standard observable, which is also a result of the findings of a research group related to the authorship of the CHEERS statement [193].

### Implications for methodological guidance and reporting standards

Although this scoping review did not aim to conduct a formal quality appraisal or assess compliance with specific reporting standards, this was a deliberate methodological decision consistent with the exploratory and inclusive purpose of a scoping review. The mapped evidence nevertheless allows for a descriptive reflection on how existing methodological guidance is operationalized in HEEs of health promotion and disease prevention in Germany. The observed heterogeneity across study designs, analytic perspectives, outcome measures, and reporting practices suggests that, while general frameworks such as CHEERS, ISPOR guidance, and the Hanover Consensus are available, their application in this domain remains inconsistent.

Several findings are particularly relevant in this regard. First, despite recommendations to adopt a societal perspective for preventive interventions with broad population effects, only 35.8% of studies incorporated such a perspective, either alone or in combination with others. Second, although uncertainty is widely acknowledged as a central concern in decision-analytic modeling, the depth and transparency of sensitivity analyses varied considerably, with probabilistic approaches and budget-impact analyses being applied less frequently than suggested by German methodological guidance. Third, the wide range of outcome measures and the frequent reliance on international cost-effectiveness thresholds highlight challenges in ensuring national relevance and comparability of results.

Taken together, these observations do not indicate a lack of methodological guidance per se, but rather highlight structural challenges in consistently applying generic economic evaluation frameworks to the specific characteristics of health promotion and disease prevention interventions. These characteristics include long-term time horizons, diffuse and intersectoral effects, behavioral outcomes, and reliance on non‑German data sources. While previous reviews have discussed methodological issues in economic evaluations of prevention and health promotion or provided narrative overviews of the literature, they did not systematically map primary HEEs within the German context or examine how such guidance is operationalized in practice. By explicitly retaining studies with variable reporting quality, the present scoping review makes these challenges visible rather than excluding them. By systematically mapping current practice, this review provides an empirical basis for identifying areas where existing guidance may require adaptation, clarification, or domain‑specific extension. Future systematic reviews focusing on specific prevention or health promotion contexts could build on this mapping by applying formal quality appraisal tools and more restrictive inclusion criteria. Such efforts could support more consistent, transparent, and decision‑relevant HEEs in the German prevention and health promotion context.

### Assessing cost-effectiveness

Regarding the reporting of cost-effective, or even cost-saving interventions, a substantial proportion of studies (85.2%) reported positive findings. Importantly, this scoping review summarizes how cost‑effectiveness is reported by authors of included studies, rather than re‑evaluating or harmonizing base‑case results across studies. However, as our review did not distinguish between cost-effectiveness in the base case versus alternative scenarios with differing parameter inputs, this observation may overrepresent positive findings and should be interpreted with caution but reflects prevailing reporting practices. Cost-effectiveness cannot be adequately answered with a simple “yes” or “no,” and the high share of positive results may indicate potential publication bias, which could complicate informed decision-making.

In terms of health outcomes, more than one-third of the studies used QALYs, making it the most commonly applied metric. Consequently, the incremental costs per QALY gained were the most frequently reported ICERs. QALYs, which combine life expectancy with health-related quality of life, are widely used to inform healthcare decision-making in countries with explicit guidance thresholds, such as the UK [19] or Canada [194]. However, the methodological and ethical limitations of QALYs are well documented [3, 194], and German decision-making does not formally rely on this metric. Instead, healthcare decisions in Germany are primarily informed by evidence from the existing literature, assessments of clinical benefit, and deliberative processes within advisory and self‑governing bodies, rather than by explicit monetary thresholds or predefined willingness‑to‑pay limits.

The use of cost-effectiveness thresholds is challenging in the German context, as no official guidance exists. Some studies have attempted to apply the National Institute for Health and Care Excellence (NICE) recommendations for the UK (20,000–30,000 GBP per QALY gained) [194], but such thresholds are highly dependent on country-specific factors, including population, context, and health states [195]. As a result, these thresholds provide only an indicative measure of cost-effectiveness and cannot be used for definitive valuation. Other authors have applied the World Health Organization (WHO) recommendation of 1–3 times the gross domestic product per capita [196], an approach criticized for its lack of direct relation to a country’s healthcare budget, technical capacity, population preferences, or social values [197, 198]. Overall, these findings highlight the need for clearer and more context‑specific methodological guidance on the interpretation and use of cost‑effectiveness results in Germany.

### Encountering uncertainties

In Germany, discussions of cost-effectiveness are typically based on opportunity costs and implicit evaluation mechanisms. Drummond et al. highlighted that, in countries without formal guidance thresholds, newly estimated ICERs can be compared with those of already funded interventions. However, this approach assumes that prior funding decisions are appropriate and that the ICERs underlying these decisions are known, which is often not the case, thereby introducing further uncertainty [3].

The German IQWiG recommends presenting ICERs as the primary outcome in HEEs, accompanied by an assessment of uncertainty derived from sensitivity analyses (SAs). A comparison with results from other HEEs addressing similar research questions is also suggested as an option [18]. Accordingly, the high proportion of modeling studies conducting SAs in our review (95.9%) indicates an awareness of uncertainty and the use of different approaches to discuss cost-effectiveness. Nevertheless, approximately three-quarters of the included studies relied on decision-analytic models based on assumptions and input values. Substantial uncertainty in their results is therefore expected. Unlike clinical trials, model-based approaches simulate hypothetical scenarios, and the reliability of their findings depends heavily on the quality of the input data; consequently, results should be interpreted with caution. IQWiG further recommends conducting budget-impact analyses (BIAs) to provide decision-makers with information on the reasonableness of interventions and potential future financial implications [18]. Despite this, only 6.2% of the studies in our review included a BIA, highlighting a gap in the practical applicability of many evaluations. Taken together, these findings highlight that, in addition to careful interpretation of cost-effectiveness, accounting for model uncertainty and conducting comprehensive sensitivity and budget-impact analyses are essential steps to support robust and policy-relevant HEEs.

### Data origin in model-based approaches

An observation made in this work was the high number of modeling studies relying on data from foreign sources. Of course, RCTs, systematic reviews, and meta-analyses represent the highest quality of evidence, yet they were classified in this review as international data sources. Regardless of this simplification, German data were rarely used as the basis for analysis, particularly in the assessment of health-related quality of life. Notably, numerous studies using international data set up reviewing boards to support the adaption of these international values to the German context. The German health care system consists of a complex structure with many independent stakeholders and no central control institution. Furthermore, data protection is a well-protected value for good reasons in Germany. Given the potential impact on the overall validity of model simulations, it can be concluded nonetheless that data availability in Germany requires further improvement. At the same time, the use of data from foreign sources is not inherently problematic and can be valuable for economic analyses in Germany, depending on the intervention and the context in which the data were generated. In such cases, careful contextualization and adaptation to the German setting are essential, and the added value of generating new country‑specific data should be critically weighed against the effort required.

### Limitations

Despite its comprehensive approach, this scoping review has several limitations that should be considered when interpreting the findings.

First, differentiating between the concepts of health promotion and disease prevention was challenging, particularly given overlaps with early treatment, rehabilitation, and disease management and missing information in the papers, respectively. For this reason, tertiary preventive interventions were excluded, as a clear delineation could not be established during the training phase.

Second, the broad research question and the design of the applied search strategy imposed inherent restrictions. While we do not claim to have captured every relevant study, we sought to mitigate this limitation by searching three established databases without time restrictions and by implementing a transparent screening process involving two experienced researchers. This process included extensive training, structured team discussions, and blinded decision-making to ensure the reliability and consistency of study selection.

Third, the heterogeneity of methodological approaches and the lack of standardized reporting in HEEs pose challenges in capturing all methodological aspects. Nevertheless, data extraction was guided by foundational literature and followed an inductive process, ensuring that the most relevant methodological elements were included. In addition, discount rates were systematically extracted for costs only, as the reporting of discounting applied to effects was inconsistent or incomplete across studies, which limited reliable descriptive analysis within the scope of this review.

Fourth, our assessment of cost-effectiveness did not distinguish between cost-effectiveness thresholds and willingness-to-pay thresholds. Although these concepts are related, they are not identical and may differ in their interpretation and application which could have introduced potential bias in the classification of interventions as cost‑effective. Given the aim of this review to provide a broad overview, this approach was deemed appropriate.

Fifth, this review did not include a formal quality appraisal of the included studies. Scoping reviews are designed to map the available literature and do not typically assess the quality of evidence [199]. Conducting a systematic quality assessment would be an important step for future research, for example, through a systematic review.

Sixth, this review was restricted to studies reporting monetary outcomes in Euros or Deutsche Mark to enhance contextual comparability within the German healthcare system; however, this may have excluded relevant evidence from studies using other currencies.

Finally, the findings of this review are specific to the German population and healthcare system. Consequently, the results may not be directly transferable to countries with different healthcare structures or social welfare systems.

## Conclusions

This scoping review provides a comprehensive overview of the landscape of HEEs in interventions for health promotion and primary and secondary prevention in Germany. By mapping a broad range of health contexts and methodological approaches, the study offers an empirical overview of how economic evidence in this field is currently generated and reported. While HEEs of preventive measures are increasingly conducted in Germany, standardized methodologies, transparency in modeling approaches and consistent outcome measures remain areas for development. The observed methodological variation and inconsistent reporting limit comparability across studies and, except in the area of vaccinations, reduce the policy usefulness of the currently available evidence for decision‑making. Existing reporting checklists, such as CHEERS, may require adaptation or supplementation to better capture the specific methodological challenges associated with health promotion and disease prevention interventions… A broader and more consistent application of transparent and context-sensitive HEE frameworks would help generate a more solid and decision-relevant evidence base, thereby better supporting stakeholders responsible for planning and prioritizing preventive and health-promoting interventions in Germany.

## Supplementary Information

Additional file 1. PRISMA Extension for Scoping Reviews (PRISMA-ScR) Checklist.

Additional file 2. Search strings adapted for each database.

Additional file 3. Detailed data extraction.

Additional file 4. Definitions for applied categories in data extraction [200–204].

## Data Availability

All the data generated or analyzed during this study are included in this published article and its supplementary information files. Metadata are available in the Open Science Framework (OSF) repository, [https://osf.io/h6749/overview].

## Abbreviations

BIA: Budget-impact analysis
CBA: Cost-benefit analysis
CCA: Cost-consequence analysis
CEA: Cost-effectiveness analysis
CEAC: Cost-effectiveness acceptability curves
CHEERS: Consolidated health economic evaluation reporting standards
CMA: Cost-minimization analysis
CUA: Cost-utility analysis
DALY: Disability-adjusted life year
DSA: Deterministic sensitivity analysis
HEE: Health-economic evaluation
HLY: Healthy life year
ICER: Incremental cost-effectiveness ratio
IQWiG: Institute for quality and efficiency in health care
ISPOR: International society for pharmacoeconomics and outcomes research
JBI: Joanna Briggs institute
LYG: Life year gained
LYS: Life year saved
NICE: National Institute for Health and Clinical Excellence
QALY: Quality-adjusted life year
PRISMA-ScR: Preferred Reporting Items for Systematic Reviews and Meta-Analyses extension for Scoping Reviews
PSA: Probabilistic sensitivity analysis
ROI: Return in Investment
SA: Sensitivity analysis
STIKO: Standing Committee on Vaccinations
VOI: Value of Information Analysis
YLL: Year of life lost
