# Supplementary material for: Health economic evaluation of health promotion and disease prevention in Germany: a scoping review
Source: Health Econ Rev. 2026 Sep 25;16:109. doi: 10.1186/s13561-026-00859-0 (PMC13615592; doi:10.1186/s13561-026-00859-0)
Supplement: Supplementary file 2 [file 13561_2026_859_MOESM2_ESM.docx]

**Additional file 2: Search strategy**

**Additional file 2.1: Search string for MEDLINE via Ovid**

Database: Ovid MEDLINE(R) ALL 1946 to March 08, 2025

Search date: March 11, 2025

Number of hits: 1.713

| Basics | humans.sh. NOT (animals.sh.) **AND** |
| --- | --- |
| Concept | **(** |
|  | **(** |
|  | (economic* OR cost*).ab,ti. adj2 (evaluation OR cal- culation OR analys* OR comparison OR health care).ab,ti. |
|  | **)** |
|  | **OR** |
|  | **(**"health economics**".**mp. OR "return on invest-  ment".mp. OR "incremental cost-effectiveness ra- tio".ab,ti. OR |
|  | "cost-minimization analysis".mp. OR "cost-of-ill- ness analysis".sh. OR "cost-utility analysis".ab,ti. OR |
|  | exp "costs and cost analysis"/ OR "cost-benefit analysis".sh. OR "cost-effectiveness analysis".sh.**)** |
|  | **) AND** |
| Context | **(** |
|  | (promotion OR program* OR prevent* OR cam- paign OR education OR precaution OR public OR rehab*).ab,ti. |
|  | **OR** |
|  | (*"health promotion"/ OR *"preventive medicine"/ OR "universal prevention".mp.OR *"primary prevention"/ OR "vaccination".sh. OR *"secondary prevention"/ OR "screening".ab,ti. OR *"tertiary prevention" OR  *"rehabilitation"/) |
|  | **) AND** |
| Population | (germany.sh.) |

**Additional file 2.2: Search string for EMBASE via Ovid**

Database: **EMBASE** 1974 to 2025 March 08

Search date: March 11, 2025 Number of hits: 1.671

| Basics | human.sh. NOT (animal.sh.) **AND** |
| --- | --- |
| Concept | **(** |
|  | **(** |
|  | (economic* OR cost*).ab,ti. adj2 (evaluation OR cal- culation OR analys* OR comparison OR health care).ab,ti. |
|  | **)** |
|  | **OR** |
|  | ( "health economics"**.**sh. OR "return on invest- ment".mp. OR "incremental cost-effectiveness ra-  tio".ab,ti. OR |
|  | "ICER".ab,ti. OR "quality adjusted life year".ab,ti,sh. OR "QALY".ab,ti. OR "budget-impact analysis".ab,ti. OR |
|  | "cost minimization analysis".sh. OR "cost of ill- ness".sh. OR "cost utility analysis".sh. OR |
|  | "cost benefit analysis".sh. OR "cost effectiveness analysis".sh.**)** |
|  | **) AND** |
| Context | **(** |
|  | (promotion OR program* OR prevent* OR campaign OR education OR precaution OR public OR re- hab*).ab,ti. |
|  | **OR** |
|  | ( *"health promotion"/ OR *"prevention"/ OR "preven- tion and control".sh. OR *"preventive medicine"/ OR "universal prevention".mp. OR *"primary prevention"/ OR "vaccination".sh. OR *"secondary prevention"/ OR "screening".ab,ti. OR *"tertiary prevention"/) |
|  | **) AND** |
| Population | ("germany".sh.) |

**Additional file 2.3: Search string for Clarivate Web of Science**

Database: Clarivate Web of Science Core Collection, Editions: All

Search date: March 11, 2025

Number of hits: 1.248

| Concept | **(** |
| --- | --- |
|  | **(** |
|  | TI=(economic* NEAR/2 (evaluation OR calculation OR analys* OR comparison OR "health care")) **OR** |
|  | AB=(economic* NEAR/2 (evaluation OR calculation  OR analys* OR comparison OR "health care")) |
|  | **)** |
|  | **OR** |
|  | **(** |
|  | TI=(cost* NEAR/2 (evaluation OR calculation OR analys* OR comparison OR "health care")) **OR** |
|  | AB=(cost* NEAR/2 (evaluation OR calculation OR analys* OR comparison OR "health care")) |
|  | **)** |
|  | **OR** |
|  | **(** |
|  | TI=("health economics" OR return on investment OR incremental cost-effectiveness ratio OR "ICER" OR "quality-adjusted life years" OR "QALY") **OR** |
|  | AB=("health economics" OR return on investment OR incremental cost-effectiveness ratio OR "ICER" OR "quality-adjusted life years" OR "QALY") |
|  | **)** |
|  | **OR** |
|  | **(** |
|  | TI=(analys* NEAR/2 (budget-impact OR cost-minimi- zation OR cost-of-illness OR cost-utility OR cost OR cost-benefit OR cost-effectiveness )) **OR** |
|  | AB=(analys* NEAR/2 (budget-impact OR cost-mini- mization OR cost-of-illness OR cost-utility OR cost OR cost-benefit OR cost-effectiveness )) **OR** |
|  | AK=(analys* NEAR/2 (budget-impact OR cost-mini- mization OR cost-of-illness OR cost-utility OR cost OR cost-benefit OR cost-effectiveness )) |
|  | **)** |
|  | **) AND** |
| Context | **(** |
|  | **(** |
|  | TI=(promotion OR program* OR prevent* OR cam- paign OR education OR precaution OR public OR re-  hab*) **OR** |
|  | AB=(promotion OR program* OR prevent* OR cam- paign OR education OR precaution OR public OR re-  hab*) |
|  | **)** |
|  | **OR** |
|  | **(** |
|  | TI=("health promotion" OR "preventive medicine" OR "universal prevention" OR "primary prevention" OR secondary prevention OR "screening" OR "tertiary prevention" OR "rehabilitation") **OR** |
|  | AB=("health promotion" OR "preventive medicine" OR "universal prevention" OR "primary prevention" OR secondary prevention OR "screening" OR "ter- tiary prevention" OR "rehabilitation") **OR** |
|  | AK=("health promotion" OR "preventive medicine" OR "universal prevention" OR "primary prevention" OR "vaccination" OR "secondary prevention" OR "screening" OR "tertiary prevention" OR "rehabilita-  tion") |
|  | **)** |
|  | **) AND** |
| Population | TS=(germany) |
