# Supplementary material for: Health economic evaluation of health promotion and disease prevention in Germany: a scoping review
Source: Health Econ Rev. 2026 Sep 25;16:109. doi: 10.1186/s13561-026-00859-0 (PMC13615592; doi:10.1186/s13561-026-00859-0)
Supplement: Supplementary file 3 [file 13561_2026_859_MOESM4_ESM.docx]

**Additional file 4: Definitions for applied categories**

| **Aspect** | **Categories** | **Definition** | **Definition source** |
| --- | --- | --- | --- |
| Context | Health Promotion | The process of enabling people to increase control over, and to improve, their health. | [1] |
|  | Primary Prevention | All measures taken before the initial manifestation of a disease to prevent its onset. | [25] |
|  | Secondary Prevention | All interventions aiming at early detection and containment of disease, where the pathological process has already begun. | [25] |
| Health topic | Dental Health | All interventions regarding the improvement of oral and dental health. | Own definition |
|  | Environmental Prevention | All interventions aiming at changing ecological, social, economic or cultural structures that contribute to the emergence and progression of diseases. | [25] |
|  | Fall Prevention | All interventions regarding the prevention of falls. | Own definition |
|  | Infection Prevention | All interventions regarding the prevention of infections. | Own definition |
|  | Lifestyle Programs | All structured interventions aimed at promoting health-enhancing behaviors through education, behavioral support or incentives. | Own definition |
|  | Mental Health | A state of mental well-being that enables people to cope with the stresses of life, realize their abilities, learn well and work well, and contribute to their community. | [200] |
|  | Nutrition | All interventions regarding health-promoting or disease preventing nutritional measures. | Own definition |
|  | Occupational Prevention | All proactive strategies and interventions implemented in workplace settings to eliminate, reduce or control exposure to occupational hazards. | Own definition based on [201] |
|  | Screening | The presumptive identification of unrecognized disease in an apparently healthy, asymptomatic population by means of tests, examinations or other procedures that can be applied rapidly and easily to the target population. | [202] |
|  | Smoking Cessation | The process of quitting tobacco use for reducing the burden of tobacco-related diseases. | Own definition based on [203] |
|  | Surgical Prevention | All interventions regarding surgical measures to prevent future diseases. | Own definition |
|  | Vaccination | The administration of a vaccine to help the immune system develop protection from a disease. | Own definition based on [204] |
| Type of HEE | Basic assumption | Classification of HEEs based on the investigated endpoint. | [20] |
|  | ICER | Incremental cost-effectiveness ratio as the incremental costs per unit of effect. | [3] |
|  | CMA | Comparison of separate cost-analyses of two or more alternative interventions with outcome measures being identical or equivalent. | [10] |
|  | CCA | Comparison of two or more alternatives under use of a structured listing of all costs and relevant outcomes associated with the interventions. No application of an overarching decision criterion. | [11] |
|  | CBA | Comparison of two or more alternatives with costs and outcomes expressed in monetary terms. | [3] |
|  | CEA | Comparison of two or more alternatives with effects expressed as natural units (e. g., lowering blood pressure in mmHg). | [5] |
|  | CUA | Comparison of two or more alternatives with effects expressed as standardized utility units. | [3] |
|  | BIA | Assessment of direct financial consequences associated with the reimbursement of health technology within a specific healthcare system. | [12] |
| Type of analysis | Modeling | All model-based approaches. By nature, these studies tend to contain uncertainties because of constraints of resources, time and information availability. | Own definition; [142] |
|  | Statistical Evaluation | All trial-based approaches with descriptive statistical evaluations. | Own definition |
|  | Cost Comparison | All trial-based approaches that analyze and contrast the expenses of two or more healthcare interventions without considering their outcomes. | Own definition |
|  | Cost Calculation | All trial-based approaches that determine the total monetary resources required for a specific healthcare service, program, or intervention. | Own definition |
| Sensitivity analyses | Univariate deterministic SA | Involves using single point estimates for each input parameter to calculate expected costs and effects under variation of these parameter values and reporting the impact on the model outputs. | [3] / Own definition |
|  | Multivariate deterministic SA | Involves the variation of more than one parameter simultaneously. | [3] / Own definition |
|  | Probabilistic SA | Characterization of parameter uncertainty by assigning probability distributions to each model parameter to reflect available evidence, often under use of Monte Carlo simulations. | [3] |
|  | CEAC | The output of a probabilistic SA presented as Cost-Effectiveness Acceptability Curve (CEAC). | [3] |
|  | Threshold analysis | Variant of one-way SA where critical values of a parameter are identified that would cause the decision about which alternative offers highest expected net benefits to change. | [3] |
|  | Scenario analysis | Construction of a series of scenarios representing a subset of potential multi-way analysis. | [3] |
| Perspective | Employer | Focus on costs and benefits directly experienced by employers or companies. | Own definition based on [3] |
|  | Patient | Focus on the costs and benefits directly experienced by the patient. | [3] |
|  | Healthcare provider | Consideration of costs and consequences relevant to a particular healthcare institution. | [3] |
|  | Societal | Aggregation of all perspectives including costs arising from resource use and productivity changes. | [3] |
|  | Third-party payer | Focus on costs estimates from the payer’s budget. | [3] |
| Cost types | Direct medical | Costs arising from resource use within the health sector linked to treatment. | Own definition based on [3] |
|  | Direct non-medical | Costs arising within health sector but not medical, e.g., transport. | Own definition based on [3] |
|  | Indirect | Costs referring to productivity losses. | [3] |
|  | Private payments | Costs borne by patients and their family members. | [3] |
| Discount | % | Following recommendation to apply discount rate to costs and benefits. | [3, 13, 20] |
| Cost-effectiveness of intervention | Yes / No | Cost-effectiveness or cost-savings as reported. | Own definition |
| Cost-effectiveness threshold | Costs / Unit | Magnitude of additional health gained per unit resources. | [195] |
| Use of foreign data | Yes / No | Use of data not originating in Germany. | Own definition |
| Study design | Standalone | Conducted independently of a clinical trial. | Own definition |
|  | Piggyback | Embedded within a clinical trial. | Own definition |
| Funding | Institutional | Reported funding by an institution. | Own definition |
|  | Industrial | Reported funding by industrial service. | Own definition |
